# Effects of the Smad4 C324Y mutation on thyroid cell proliferation

**DOI:** 10.3892/ijo.2013.1908

**Published:** 2013-04-17

**Authors:** SONIA D’INZEO, ARIANNA NICOLUSSI, FRANCESCO NARDI, ANNA COPPA

**Affiliations:** 1 Departments of Experimental Medicine, Sapienza University of Rome, I-324-00161 Rome, Italy; 2 Radiological Sciences, Oncology and Anatomical Pathology, Sapienza University of Rome, I-324-00161 Rome, Italy

**Keywords:** transforming growth factor-β, Smad4, CREB, cyclin D1, papillary thyroid carcinoma

## Abstract

Smad4 is a key mediator of the transforming growth factor-β (TGF-β) superfamily that is involved in the control of cell proliferation and differentiation. We recently demonstrated that a Smad4 mutation, Smad4 C324Y, isolated from nodal metastases of papillary thyroid carcinoma, causes an increase of TGF-β signaling, responsible for the acquisition of transformed phenotype and invasive behaviour in thyroid cells stably expressing this mutation. In this paper, we demonstrate that the stable expression of Smad4 C324Y mutation in FRTL-5 cells is responsible for TSH-independent growth ability. Our data show that the Smad4 C324Y mutation interacts with P-Smad3 more strongly than Smad4 wt, already in basal condition; this interaction is responsible for TGF-β signaling and PKA activation that, in turn, determines an increased phosphorylation of CREB, necessary for the mitogenic actions of TSH. The expression of cyclin D1 also increases in all cells overexpressing the Smad4 C324Y mutation. All together, these data demonstrate that Smad4 C324Y mutation, interacting with the PKA pathway, gives cells the ability to proliferate independently from TSH.

## Introduction

Smad4 is a key mediator of the transforming growth factor-β (TGF-β) superfamily that is involved in the control of cell proliferation, differentiation and apoptosis (1,2). Smad4 oligomerizes with the R-Smads (Smad2 and 3), phosphorylated by the type I TGF-β receptor (TβRI), to form transcriptional complexes Smad2/3-Smad4 that translocate to the nucleus, where they bind to the promoters of target genes, activating or repressing their transcription (3,4).

TGF-β is normally expressed and secreted in epithelial follicular thyroid cells, where it controls the differentiated phenotype, inhibits iodide trapping (5,6), and thyroglobulin synthesis (7), exercising some of these effects through Smad signaling (8,9). TGF-β is also the negative regulator of thyrocyte proliferation and is able to antagonize the mitogenic effects of the main growth factors in thyroid cells of rat (7,10–12), of porcine (13) and of human (14), delaying progression during the mid-late G1 phase (12,15).

Impairment of the TGF-β signaling at the level of Smad genes is common in human carcinomas. Absent or decreased expression of Smad4 has been demonstrated in various cancers, including pancreatic, colorectal, head and neck (16,17), and, more recently, in papillary thyroid carcinomas (PTCs) (18), suggesting that the TGF-β signaling functions as a tumor suppressor. On the other hand, TGF-β can exhibit tumor-promoting effects as observed in prostate and skin cancer progression (19,20) and in papillary thyroid carcinomas (21). We recently demonstrated that a Smad4 mutation, Smad4 C324Y, isolated from nodal metastases of papillary thyroid carcinoma, causes an increase of TGF-β signaling responsible for the acquisition of transformed phenotype and invasive behaviour in thyroid cells stable expressing this mutation (22). The TGF-β inhibitory growth response is also reduced in these cells, this finding is consistent with the observation that when Smad4 C324Y mutation is expressed in thyroid cells it exerts a clear pro-oncogenic function. The fine regulation of thyrocytes growth and differentiation reflects a critical balance between the promotion and suppression of cell division. TSH has been shown to stimulate, through the activation of its receptor, more than one signal transduction pathway, most notably the adenylcyclase/cAMP (cyclic adenosine monophosphate) pathway. cAMP seems to account for the mitogenic effects of TSH in human thyroid cells, mediated by the activation of cAMP-dependent protein kinase A (PKA) (23,24). Therefore, the FRTL-5 cell line, that maintains *in vitro* all the markers of thyroid cell differentiation, represents an excellent model to study the mechanism regulating thyroid cell proliferation, because they require stimulating factors like TSH or insulin for their growth.

In this study, we demonstrated that the stable expression of Smad4 C324Y mutation in thyroid cells is responsible of TSH independent growth ability, without any modulation of thyroid specific genes, like thyroglobulin (TG). This response is caused by an increase in oligomerization of Smad4 with Smad3, responsible, in turn, for an increased phosphorylation of CREB, necessary for the mitogenic actions of TSH.

## Materials and methods

### Reagents and constructs

Dulbecco’s modified Eagle’s medium (DMEM), Coon’s modified Ham’s F-12 medium, PBS, bovine serum (BS), trypsin-EDTA, L-glutamine 100X (200 mM), the six-hormone mixture (6H) containing: TSH (10 mU/ml), insulin (10 *μ*g/ml), hydrocortisone (10^−8^ M), transferrin (5 *μ*g/ml), glycyl-L-histidyl-L-lysine acetate (10 ng/ml), and somatostatin (10 *μ*g/ml) were purchased from Sigma-Aldrich (St. Louis, MO, USA). The human recombinant TGF-β1 isoform was purchased by PeproTech (Rocky Hill, NJ, USA). Phenilmethylsulfonil fluoride (PMSF), protease cocktail inhibitor containing 1 mg/ml leupeptin, 2.5 mg/ml aprotinin, 1 mg/ml benzamide hydrochloride and phosphatase inhibitor cocktail were purchased from Sigma-Aldrich. Antibodies to Green Fluorescent Protein (GFP FL), cyclin D1 (C20), cyclin D1 (H-295) and β-actin (C4) from Santa Cruz Biotechnology (Santa Cruz, CA, USA). Antibodies to the phosphorylated form of Smad3 (P-Smad3) from Cell Signaling Technology (Beverly, MA, USA), to P-CREB (phospho S133) and CREB from Abcam (Cambridge, UK). Horseradish peroxidase conjugated secondary antibodies were purchased from Sigma-Aldrich. Transfections were performed using lipofectin kit provided by Lipofectamine Plus Gibco-BRL, Life Technologies (Rockville, MD, USA). Stable clones were obtained by selection with geneticine G418 (Invitrogen-Life Technologies, Carlsbad, CA, USA). GFP-tagged Smad4 constructs were obtained subcloning the human *SMAD4* gene in Clontech (Palo Alto, CA, USA) pEGFPC3 vector, which allows in frame fusion to the C terminus of GFP.

### Cell cultures

The FRTL-5 (ATCC #8305) were kindly provided by Dr L.D. Kohn (NHI, Bethesda, MD). This cell line, diploid between their 5th and 25th passage, maintains the functional characteristics of iodide uptake, thyroglobulin synthesis and cyclic nucleotide metabolism over prolonged periods of culture and doubling time of approximately 36 h (25). These cells were grown as previously described (8) in W/O supplemented with 5% calf serum and six-hormone mixture (6H) containing: TSH (10 mU/ml), insulin (10 *μ*g/ml), hydrocortisone (10^−8^ M), transferrin (5 *μ*g/ml), glycyl-L-histidyl-L-lysyne acetate (10 ng/ml), and somatostatin (10 *μ*g/ml). Clones obtained by stable transfection of the FRTL-5 cells with the expression vector pEGFPC3 containing the human Smad4 cDNA wt or mutated, tagged with GFP, or with pEGFPC3 empty vector, were grown in F-12 Coon’s modification medium supplemented with 5% bovine serum and 6H mixture in presence of 500 *μ*g/ml of G418, as previously described (22). MDA MB468 (26), breast cancer cell lines purchased from American Type Culture Collection (ATCC, Rockville, MD), were grown in DMEM supplemented with 10% BS. All cells were maintained in continuous monolayer cultures at 37°C and 5% CO_2_, expanded up to 70–80% confluent, treated or not with human recombinant TGF-β1 (10 ng/ml) and then employed for the experiments as described below.

### RNA isolation and analysis

RNA was extracted using Tri Reagent (Sigma-Aldrich), following the manufacturer’s instructions. Using 1 *μ*g RNA, cDNAs were synthesized using MuLV Reverse Transcriptase (Applied Biosystems, NJ, USA) and random primers (Roche, Mannheim, Germany) according to the manufacturer’s instructions. The primers used in the amplification of rat TG (TG forward 5′-TGCCCACCCAGAATCAAGGAAC-3′, reverse 5′-TGAAGCCAAAGGTACCCACAACTG-3′) and rat GAPDH, as internal control (GAPDH forward 5′-TTCACCACCATGGAGAAGGCT-3′, reverse GAPDH 5′-ACAGCCTTGGCAGCACCAGT-3′) were designed to cross intron-exon junctions. Each experiment was repeated three times using different total RNA extracts. TG and GAPDH bands were analyzed using Bio-Rad Laboratories software. Data were collected in terms of average intensity of bands of TG amplicon per average intensity of GAPDH.

### Proliferation assays

The cell proliferation was assessed by cell-counting. Briefly, 10×10^4^ cells were seeded into 35-mm plate and left overnight at 37°C in a humidified incubator with 5% CO^2^. After a starvation in 4H/0.5% BS medium for 24 h, culture medium was changed in F-12 Coon’s modification medium supplemented with 0.5% BS and 5H mixture (6H mixture without TSH or insulin). Growth curves were obtained counting the cells at time zero (T0), 24, 48, 72 and 96 h. Cells were counted three times by two independent investigators. Inter-observer variation was below 5%. Values represent mean of triplicate determination ± SD of three experiments.

### Cell cultures and transient transfection analysis

MDA MB468 (26) were transiently transfected with pEGFPC3-Smad4 wt or pEGFPC3-Smad4 C324Y using lipofectin technique following the manufacturer’s instructions. After 24 h incubation, transfected cells were used in immunoprecipitation experiments and western blot assays.

### Immunoblot analysis and immunoprecipitation

Subconfluent cells, transfected or not, were treated or untreated with 10 ng/ml of TGF-β1 for 60 min. Protein extracts were obtained using ice-cold TNE extraction buffer (50 mM Tris-HCl pH 7.8, 150 mM NaCl, 1 mM EDTA, 1% Triton X-100) supplemented with 1 mM PMSF, protease and phosphatase cocktail inhibitors. Protein lysates (60 *μ*g) were subjected to immunoblot analysis as previously described (27) using primary antibodies to GFP FL (1:500), P-CREB (1:2,000), CREB (1:1,000), cyclin D1 (1:1,000), P-Smad3 (1:500) and β-actin (1:5,000). Membranes were, then, incubated with anti-rabbit (1:50,000) or anti-mouse (1:10,000) horseradish peroxidase-conjugated secondary antibodies. The western blots were revealed by chemiluminescence using the Super Signal kit from Pierce (Rockford, IL, USA) according to the manufacturer’s instructions and visualized on CL-Xposure Film (Pierce). For immunoprecipitation experiments, 1 mg of total protein extracted as previously described, was precleared with protein-A Sepharose CL-4B (GE Healthcare, Uppsala, Sweden) beads and then immunoprecipitated with anti-GFP polyclonal antibody (Sigma-Aldrich). Immunocomplexes, aggregated with 50 *μ*l of protein-A Sepharose CL-4B, were washed four times with 1 ml of buffer. The pellets were boiled in Laemmli buffer for 5 min, and the proteins were resolved under reducing conditions by 8% SDS-PAGE and subjected to immunoblot analysis as previously described (27) using primary antibodies to P-Smad3 and GFP FL. P-CREB, cyclin D1 bands were analyzed using Bio-Rad Laboratories software. Data, obtained from three different protein extracts, were collected in terms of average intensity of bands of each protein per average intensity of bands of CREB or β-actin.

### Immunohistochemistry analysis

Thyroid tissue sections of lymph node metastasis of PTC from which derives the C324Y mutation of Smad4 and 3 lymph node metastases of PTC without mutation, collected at the Anatomic Pathology of Department of Experimental Medicine of Sapienza University of Rome from 1996 to 2001, were studied using the catalyzed signal amplification protocol (Dako A/S, Glostrup, Denmark) (28). Slides were incubated in a humidified chamber overnight at 41°C with 1:100 dilution of polyclonal serum to cyclin D1 (H-295). The peroxidase-based LSAB2 Detection kit (Dako A/S), followed by haematoxylin counterstaining, has been used to visualize the reactions. Written informed consent was obtained from each patient according to Helsinki Declaration and approved by the local ethics committee.

### Statistical analysis

All statistical analyses were performed using JMP Software purchased by Statistical Discovery SAS Institute. Data were analyzed by Student’s t-test (P<0.05, statistical significance).

## Results

### Proliferation and differentiation in Smad4 C324Y stable clones

**Figure 1:**
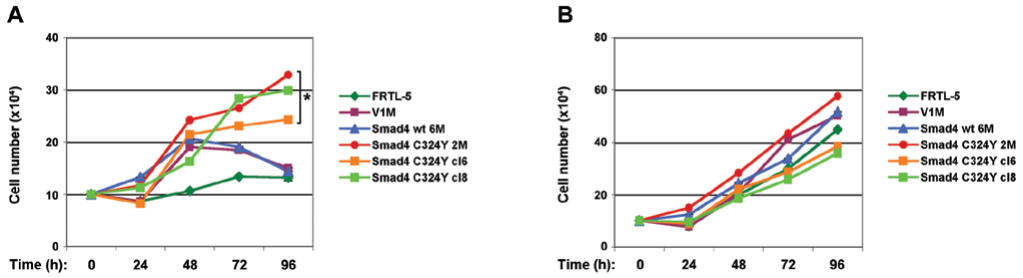
Smad4 C324Y stable clones proliferation in absence of TSH or insulin. (A and B) After starvation in 4H/0.5% BS medium for 24 h, FRTL-5 and stable clones (10×10^4^) were grown in F-12 Coon’s modification medium supplemented with 0.5% BS and 5H mixture, 6H mixture without (A) TSH or (B) insulin. Growth curves have been obtained counting the cells at time zero (T0), 24, 48, 72 and 96 h. Cells were counted three times by two independent investigators. Inter-observer variation was below 5%. Values represent mean of triplicate determination ± SD of three experiments [^*^ indicates a statistical significance (Student’s t-test, P<0.05) of Smad4 C324Y clones vs. control cells].

**Figure 2:**
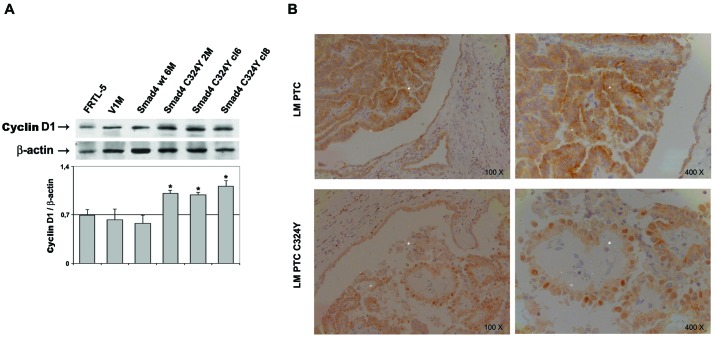
Expression of cyclin D1. (A) Whole protein lysates (60 *μ*g/lane) from FRTL-5, V1M and Smad4 stable clones (wt 6M, C324Y 2M, C324Y cl6, C324Y cl8) were analyzed by western blotting using an antibody against cyclin D1. Densitometric evaluation of the cyclin D1 signals was performed normalizing to the levels of β-actin [^*^ indicates a statistical significance (Student’s t-test, P<0.05) of Smad4 C324Y clones vs. control cells]. (B) Immunohistochemistry, using antibody against cyclin D1, was performed in lymph node metastasis of PTC from which derives the C324Y mutation of Smad4 and in a group of 3 lymph nodal metastases of PTC without mutation. Original magnification, ×100 (panels on the left) and ×400 (panels on the right).

The main positive regulators of thyroid growth and function are TSH and insulin (29). To investigate if Smad4 C324Y interferes with cell growth mediated by TSH or insulin, growth curve assays have been performed in FRTL-5 stable transfected with the expression vector pEGFPC3 containing wt or mutated Smad4 cDNAs, tagged with GFP, as previously described by D’Inzeo *et al*(22). Stable clones C324Y 2M, C324Y cl6, C324Y cl8, V1M, Smad4 wt 6M clones and FRTL-5, were grown in absence of TSH or insulin. Cells were starved in 4H/0.5% BS medium for 24 h, plated (10×10^4^ cells/plate) and counted after 24, 48, 72 and 96 h in medium supplemented with 0.5% bovine serum and 5H mixture (6H mixture without TSH or insulin). The results shown in Fig. 1A demonstrated that all Smad4 C324Y clones were able to proliferate in absence of TSH at all time intervals examined. The growth rate observed at 96 h was statistically significant (P<0.05) compared to all control cells (Fig. 1A). However, no significant differences were observed in proliferation of C324Y clones grown in absence of insulin, with respect to all controls, at all times examined (Fig. 1B). These data demonstrated that the expression of Smad4 C324Y mutation exerted a positive effect on thyroid proliferation in a TSH-independent manner. Deregulated expression of cyclin D1 is frequently an early step in neoplastic transformation in various human cancers including thyroid tumors (30). To evaluate whether the TSH-independent proliferation observed in Smad4 C324Y cells, modifies the cyclin D1 expression, we performed western blot analysis on total lysates obtained from all clones and control cells, starved in 4H/0.5% BS medium for 24 h. As shown in Fig. 2A, the presence of Smad4 C324Y mutation determined a statistically significant (P<0.05) increase of cyclin D1 expression level (about 1.7-fold induction) with respect to control cells (Fig. 2A). In addition, the lymph nodal metastasis of PTC, from which the Smad4 C324Y mutation has been isolated, was examined for cyclin D1 expression by immunohistochemistry and compared to a group of 3 lymph nodal metastases of PTC without mutation. Our results showed a strong nuclear localization of cyclin D1 in the C324Y lymph nodal metastases with respect to that of not mutated samples (Fig. 2B). Therefore, these data demonstrate that the expression of Smad4 C324Y in thyroid cells might contribute to provide the cells with a growth advantage, outlining the important role of Smad4 in thyroid carcinogenesis.

**Figure 3:**
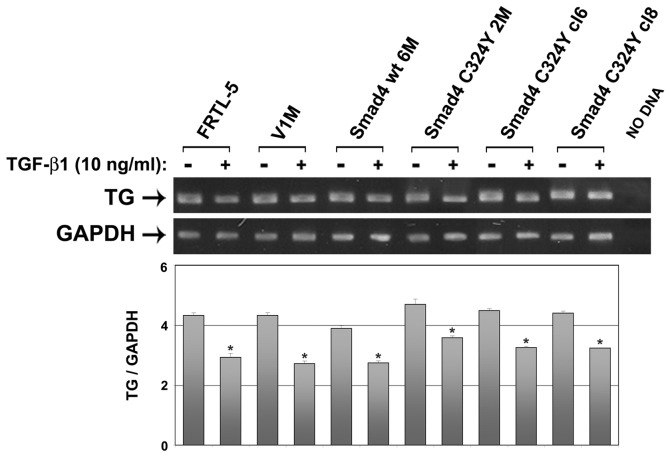
TG expression in Smad4 C324Y stable clones. Semiquantitative reverse transcription-polymerase chain reaction (RT-PCR) was performed on cDNAs obtained from FRTL-5, V1M, Smad4 wt 6M and Smad4 C324Y clones, treated or not with 10 ng/ml TGF-β1 for 24 h. Data reported represent the mean of three independent experiments. Histogram of densitometric analysis of TG bands normalized to the GAPDH levels [^*^ indicates a statistical significance (Student’s t-test, P<0.05) of treated vs. untreated cells].

Thyroglobulin represents one of the main differentiation genes in thyroid cells, whose expression is modulated by TGF-β in physiological condition (8). By reverse transcription-polymerase chain reaction (RT-PCR) performed on the same clones (FRTL-5, V1M, Smad4 wt 6M, Smad4 C324Y clones), treated or not with 10 ng/ml TGF-β1 for 24 h, we demonstrated that the overexpression of Smad4 C324Y mutation is not associated with significant modification of TG expression levels in basal condition, neither does it modify the response to TGF-β treatment (Fig. 3).

### Smad3/Smad4 C324Y oligomerization

**Figure 4:**
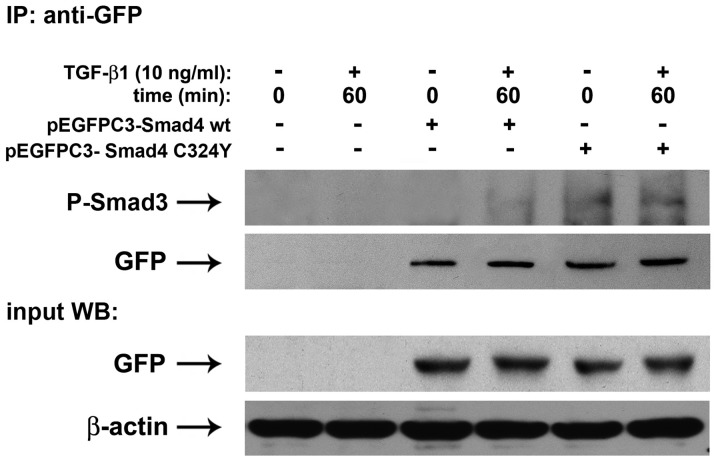
Smad3/Smad4 C324Y oligomerization. One milligram of total lysates was obtained by MDA MB468, untransfected and transiently transfected with pEGFPC3-Smad4 wt or pEGFPC3-Smad4 C324Y, untreated or treated with 10 ng/ml TGF-β1 for 60 min. Proteins were immunoprecipitated with anti-GFP antibody, resolved under reducing conditions by 8% SDS-PAGE and subjected to immunoblot analysis using primary antibody to P-Smad3 (1:500) and GFP (1:500). Transiently transfection and equal loading were verified using primary antibodies to GFP and β-actin.

We already demonstrated that Smad4 C324Y mutation determines an increase of oligomerization of Smad4 with R-Smad, Smad2 and a lengthening of nuclear localization, responsible for an increase of TGF-β signaling (22). To investigate the effect of Smad4 C324Y in the formation of complexes with P-Smad3, immunoprecipitation experiments with anti-GFP antibody were performed on lysates from MDA MB468, cell line that lacks endogenous SMAD4 because of homozygous deletion of the *SMAD4* gene (26), untransfected and transiently transfected with pEGFPC3-Smad4 C324Y or transfected with pEGFPC3-Smad4 wt, untreated or treated with 10 ng/ml TGF-β1 for 60 min. These immunoprecipitates were probed with an antibody that recognized P-Smad3. The results in Fig. 4 show that in cells overexpressing Smad4 C324Y, the interaction with P-Smad3 was stronger than that observed in cells over expressing Smad4 wt, both in basal condition and after 60 min of treatment (Fig. 4). These results allow us to conclude that the presence of Smad4 C324Y was responsible for the formation of transcriptional complexes, able to maintain a longer activation of TGF-β signaling.

### CREB levels in Smad4 C324Y stable clones

**Figure 5:**
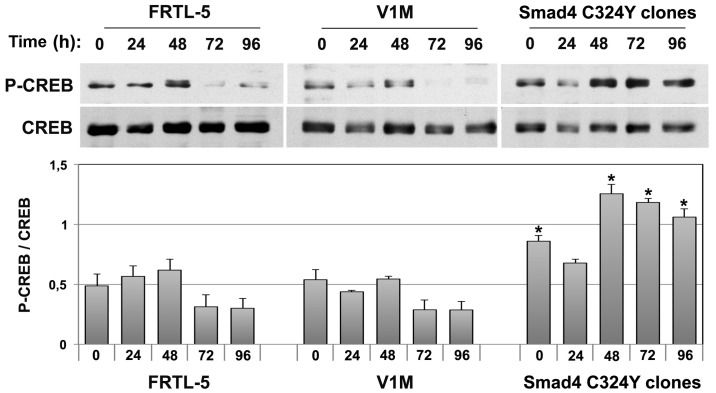
CREB phosphorylation level in Smad4 C324Y stable clones. Total lysates were obtained from FRTL-5 and stable clones starved in 4H/0.5% BS medium for 24 h (T0) and cultured for 24, 48, 72 and 96 h in medium without TSH. Protein extracts (60 *μ*g/lane) were analyzed by western blotting, using an antibody against P-CREB (1:2,000) and CREB (1:1,000) as equal loading control. Histograms of densitometric analysis of the P-CREB bands normalized to the respective unphosphorylated form. Values represent mean of triplicate determination ± SD of three experiments [^*^ indicates a statistical significance (Student’s t-test, P<0.05) of Smad4 C324Y clones vs. control cells].

It has been demonstrated that the P-Smad3/Smad4 complex interacts with and activates the PKA, resulting in CREB phosphorylation and activation of downstream target genes (31). To verify the influence of Smad4 C324Y mutation on CREB phosphorylation, we performed western blot analysis on total cell lysates obtained from cells starved in 4H/0.5% BS medium for 24 h (T0) and cultured for 24, 48, 72 and 96 h in 5H-TSH/0.5% BS medium. As shown in Fig. 5, P-CREB was significantly (P<0.05) increased already in basal condition in Smad4 C324Y clones with respect to FRTL-5 and V1M control cells (1.7- and 1.6-fold induction, respectively); this augmentation was still evident at 48, 72 and 96 h (P<0.05) (Fig. 5). Given the role of CREB in the control of proliferation of thyroid cells, an increased level of its phosphorylation could contribute to the higher proliferative capacity of the clones overexpressing Smad4 C324Y.

## Discussion

TGF-β signaling plays a dichotomous role in tumor progression and, depending on the cancer type and tumor stage, may act early as a tumor suppressor and in late-stages as a pro-metastatic pathway. Smad4 is a key signal transducer of the TGF-β superfamily that controls a broad range of cellular processes ranging from proliferation to differentiation and apoptosis (1,2). Smad4 plays an important role in human physiology, and its mutations were found with high frequency in a wide range of human cancers (16,17,32). We have recently demonstrated that a Smad4 mutation, Smad4 C324Y, isolated from nodal metastases of papillary thyroid carcinoma (33), determines a significant increase of TGF-β signaling, causing the acquisition of transformed phenotype and invasive behaviour when stably expressed in thyroid cells (22).

In this study, we focus on the effects of Smad4 C324Y mutation on thyroid cell proliferation and differentiation. Abnormal thyroid cell proliferation has a very important role in human diseases. Its deregulation causes goiter, thyroid adenomas, and carcinomas or primary hypothyroidism resulting from hypoplasia (34). The main regulators of thyroid growth and function are TSH and insulin, which recognize both insulin- and IGF-I receptors. In FRTL-5 cells, that retain most of the features of differentiated follicular thyroid cells, these growth factors can produce a dose-dependent increase in DNA synthesis and cell proliferation (29). Many studies demonstrated that TSH is the main mitogenic factor in thyroid (35,36), although others have shown that TSH exerts only a priming effect, making the cell more competent to progress into G1 phase in response to insulin/IGF-I alone (37,38). The view that in FRTL-5 cells, proliferation and DNA synthesis are synergistically activated by TSH and insulin/IGF-I is almost unanimously accepted (39,40). In this paper, we demonstrate that when Smad4 C324Y is expressed, all clones were able to proliferate in TSH independent manner, while the growth rate of cells growing without insulin is comparable to that of control cells. These data let us to state that this Smad4 mutation affects only the proliferative response to TSH. In FRTL-5, the mitogenic effects of TSH are mainly or totally mediated by cAMP and require PKA activity. Binding of TSH to its receptor (TSHR) results in an increase of cAMP intracellular concentration, which activates PKA to phosphorylate CREB and other substrates, leading to gene transcription modification, associated with both proliferation and differentiation (29). Here we demonstrated that all Smad4 C324Y clones present a significant increase of CREB phosphorylation compared to control cell lines already in basal condition. Given the role of CREB in the control of proliferation of thyroid cells, an increased level of its phosphorylation could be responsible of the proliferative behaviour of the clones overexpressing the mutation of Smad4.

A cyclic AMP-responsive element (CRE), located upstream of the cyclin D1 mRNA start site, integrates mitogenic signals that target the CRE-binding factor CREB, which can recruit the transcriptional coactivator CREB-binding protein (CBP). Transcriptional activation of the cyclin D1 gene is a key step in cell proliferation: cyclin D1 is normally expressed during G1 and regulates the transition from G1 to S phase (30). Deregulated expression of cyclin D1 is frequently an early step in neoplastic transformation in various human cancers including thyroid tumors (30,41–44). Interestingly, several studies have also reported correlations between cyclin D1 overexpression and poor clinical prognostic characteristics, including advanced tumor stage, tumor recurrence, and metastatic spread in papillary thyroid (45,46). Although the copy number of cyclin D1 gene is amplified in a number of human neoplasms, neither major genetic alterations nor amplification of this gene has been found in thyroid cancers, but mainly a strong nuclear cyclin D1 localization (47,48). This finding allow us to hypothesize that overexpression of cyclin D1 probably could be a secondary effect, induced by other genetic alterations in thyroid cancer (30). The expression of cyclin D1 is increased in all cells overexpressing the Smad4 C324Y mutation *in vitro* and it is strongly nuclear in lymph node metastasis of PTC from which derives the C324Y mutation of Smad4.

An important interaction has been demonstrated between the TGF-β and PKA signaling pathways, mediating several physiological responses elicited by TGF-β including CREB activation. Activated Smad3/Smad4 complexes that bind the regulatory subunit of PKA, independently of cAMP levels, mediate this interaction. In this way, the catalytic subunit of PKA is released, resulting in phosphorylation of CREB and activation of downstream target genes (31). Our data show that Smad4 C324Y mutation has a higher capacity to interact with P-Smad3 than with Smad4 wt, already in basal condition, thus attesting a strong activity of TGF-β signaling, responsible also for PKA activation. All together, these data support the direct involvement of Smad4 C324Y mutation in the increase of P-CREB levels and clarify the different capacity of growth observed in these clones in absence of TSH.

Despite hyper-activation of TGF-β signaling causes a reduction of TG gene expression (8,24,49), we demonstrated that the overexpression of Smad4 C324Y mutation does not determine a different behaviour in stable clones either in basal condition or after TGFβ-treatment. We think that the point mutation C324Y, localized in the MH2 domain of Smad4 protein, does not interfere with the control of TG expression. Collectively, these data demonstrate that Smad4 C324Y mutation, interacting with PKA pathway, gives the cell the ability to proliferate independently of TSH. Therefore, we conclude that TGF-β signaling plays a key role in thyroid carcinogenesis and can be considered as a new prognostic and therapeutic target for thyroid cancer.
